# *N*-Acetylcysteine Attenuates Cisplatin-Induced Acute Kidney Injury by Inhibiting the C5a Receptor

**DOI:** 10.1155/2019/4805853

**Published:** 2019-04-14

**Authors:** Shuai Huang, Jian You, Kun Wang, Yueqiang Li, Ying Zhang, Haotian Wei, Xinjun Liang, Yanyan Liu

**Affiliations:** ^1^Department of Nephrology, Division of Internal Medicine, Tongji Hospital, Tongji Medical College, Huazhong University of Science and Technology, Wuhan, Hubei, China; ^2^Daping Hospital, Army Medical University (The Third Military Medical University), Chongqing, China; ^3^Wuhan Forth Hospital, Puai Hospital, Tongji Medical College, Huazhong University of Science and Technology, Wuhan, Hubei, China; ^4^Department of Medical Oncology, Hubei Cancer Hospital, Tongji Medical College, Huazhong University of Science and Technology, Wuhan, Hubei, China

## Abstract

N-acetylcysteine has been widely used as a nutritional supplement and drug in humans for its antioxidant properties. The complement activation fragment C5a is a strong proinflammatory molecule that mediates cell adhesion, chemotaxis, and the complex biological functions. However, the effect of NAC on the C5a, and the relationship of those two with cisplatin-induced acute kidney injury are unknown. In cisplatin induced AKI mouse model, mice with NAC administration had a marked improvement in renal function (BUN and Cr), decreased pathological damage, reduced inflammation, and alleviated renal oxidative stress. Furthermore, C5a and C5aR expression in the cisplatin-treated group was notably increased compared with the control group, and this increase could be significantly inhibited by NAC. In addition, neutrophils coexpressed distinctly with C5aR, and the number of infiltrating neutrophils (MPO^+^ly6G^+^) and inflammatory factors decreased with NAC treatment in the cisplatin-treated group. Overall, these data demonstrate that NAC could ameliorate cisplatin-induced nephrotoxicity in mice and the protective effects may be conducted by inhibiting the activation of kidney inflammation and the complement system.

## 1. Introduction

Acute kidney injury (AKI) is an abrupt loss of the kidney function in a short period of time, with a sharp increase in the blood creatinine levels. The acute kidney injury network (AKIN) reported that the morbidity of acute kidney injury is 2–7% in inpatients and even as high as 5–10% in ICU patients [1, 2]. Nowadays, the morbidity of drug-associated kidney injury is rising and already accounts for about 20% of all AKI cases, making it the most important cause of AKI [3].

Cisplatin is one of the most common drugs that induce AKI [4]. Cisplatin, also known as cis-Dichlorodiammine platinum (II), is a chemotherapy drug used to treat a number of solid tumors, but its renal toxicity greatly limits its use [5]. Although most antitumor alkylating agents cause DNA damage in specific, rapidly growing cells, cisplatin (an alkylating-like agent) can also cause severe damage to the proximal renal tubules. The mechanism of cisplatin-induced AKI is complex and involves many interrelated factors, mainly including the accumulation of cisplatin mediated by transmembrane transport, DNA damage, mitochondrial dysfunction, oxidative stress, inflammatory reaction, and activation of the apoptosis pathway [6, 7].

N-Acetylcysteine (NAC), a precursor of glutathione, has been used as a mucolytic drug for treating cystic fibrosis for more than 40 years [8]. In the 1970s, NAC was used to treat acetaminophen poisoning. Until 1980, it was used for patients with chronic diseases with decrease the glutathione while increasing oxidative stress (like the alcoholic liver disease) [9–11]. In addition to its antioxidation effect, NAC has an anti-inflammatory effect [11–15].

In acute inflammation, complement system, and inflammatory cells play an important role. The C5a complement activation fragment is a strong proinflammatory molecule that, on binding to the C5a Receptor (C5aR), mediates cell adhesion, chemotaxis, and complex biological functions [16, 17]. C5aR is a class A transmembrane G-protein-coupled receptor (GPCR) and is widely expressed on the surfaces of polymorphonuclear neutrophils (PMN), mononuclear cells, and nonimmune cells, which exist in many tissues, such as kidney, liver, spleen, and heart tissues [18–21]. This paper studies the protective effect of NAC against the nephrotoxicity of cisplatin and discusses the relationship between the protective effect of NAC and the complement C5a.

## 2. Materials and Methods

### 2.1. Animals

All animal protocols were approved by the Institutional Animal Care and Use Committee at the Tongji Medical College, Huazhong University of Science and Technology. Eight-week-old male C57BL/6 mice were obtained from Hua Fukang Experimental Animal Center (Beijing, China). The mice were housed in the animal facility of the Tongji Medical School and fed with water and food ad libitum. After one week of acclimation, the mice were randomly divided into 4 groups (n =8 per group): (i) Sham group, in which mice were administered saline for 3days by intraperitoneal (i.p.) injection; (ii) Cisplatin group, in which mice were administered 22 mg/kg body weight cisplatin (1.0 mg/ml solution in sterile normal saline) by a single intraperitoneal injection and then injected saline intraperitoneally for another 3 days; (iii) N-Acetylcysteine group, in which mice were administered NAC for 3 consecutive days by i.p. injection (50mg/kg); (iv) Cisplatin + Acetylcysteine group, in which mice were administered cisplatin (22mg/kg) by a single i.p. injection and then injected NAC intraperitoneally for 3 consecutive days. The mice were sacrificed; then kidneys and blood were collected for further experiment.

### 2.2. Assessment of Renal Function

Blood was centrifuged at 1450 g for 10 min and the serum supernatant was obtained. Blood urea nitrogen (BUN) and serum creatinine (Cr) levels were determined in the clinical laboratory of Tongji Hospital for evaluation of renal function.

### 2.3. Oxidation Index Detection

Glutathione (GSH), malondialdehyde (MDA), and superoxide dismutase (SOD) commercial assay kits were all provided by Nanjing Jiancheng Bioengineering Research Institute (Nanjing, China). The kidney tissues were homogenized with 0.9% NaCl and centrifuged (16,500 g, 30 min, 4°C). The supernatants were separated and the levels of MDA, GSH, and SOD were measured according to the manufacturer's protocol.

### 2.4. Histological Analysis

The kidneys from mice were fixed in 4% formalin and then embedded in paraffin. The 4 *μ*m renal paraffin sections were stained with periodic acid Schiff (PAS) and subsequently evaluated by two investigators who were blinded to our treatments. The five-point quantitative scale was used, containing the degree of tubular necrosis, hemorrhage, and cast formation as follows: 0, < 10%; 1, 10-25%; 2, 25-50%; 3, 50-75%, and 4, 75-100%.

### 2.5. Immunohistochemical Analysis

After antigen retrieval, inactivating endogenous peroxidase, and the paraffin sections were incubated with primary antibodies of C5aR (1:100; Proteintec, Chicago, IL, USA) at 4°C overnight. The sections were then incubated with goat anti-rabbit IgG (1:100; Wuhan Saiweier Biotechnology Co., Ltd.) at room temperature for 30 min, followed by reaction with diaminobenzidine (OriGene Technologies, Inc.). The images of stained sections were acquired by a Nikon DXM 1200 digital camera and the expression level of those proteins was analyzed with Imagepro-Plus.

### 2.6. Immunofluorescence

The paraffin-embedded renal sections were incubated with the following primary antibodies at 37°C for 2 hours: Ly6G (1:100; Abnova, San Diego, CA, USA) and C5aR (1:50; Proteintech, Chicago, IL, USA). Then, the slides were washed with PBST and incubated for 45 at 37°C minutes with secondary antibodies: FITC-conjugated goat anti-rat IgG and Cy3-conjugated goat anti-rabbit IgG (1:200; Wuhan Saiweier Biotechnology Co., Ltd.). After washing, the cell nucleus was dyed by DAPI (2 *μ*g/ml; cat. no. D9542; Sigma-Aldrich; Merck KGaA) at room temperature for 5 minutes. Sections were observed under Nikon fluorescence microscope (Nikon ECLIPSE TE2000-U, Tokyo, Japan) and the images were obtained by this machine.

### 2.7. Western Blot Analysis

The renal tissue was fixed with RIPA lysis buffer which contains cocktail protease inhibitors (Wuhan Saiweier Biotechnology Co., Ltd., Wuhan, China) to extract the total proteins for 30 minutes on the ice and then centrifuged at 16,500 g for 30 min at 4°C to collect the supernatant. Protein concentration was quantified by a BCA protein assay kit (Beyotime Institute of Biotechnology, Shanghai, China). A total of 50 *μ*g protein sample was separated by 10% SDS-PAGE and subsequently transferred to nitrocellulose membranes (EMD Millipore, Billerica, MA, USA). Following blockage of nonspecific antigen with 5% bovine serum albumin (BSA; Sigma-Aldrich; Merck KGaA) for 1 hour at room temperature, C5aR primary antibody (Proteintech, Chicago, IL, USA) was used at a dilution of 1:500, and GAPDH (Proteintech, Chicago, IL, USA) primary antibodies were used at a dilution of 1:3000. The membranes containing tissue protein were incubated overnight at 4°C. After washing with TBST, the bands were incubated with goat anti-rabbit or goat anti-mouse secondary antibodies (1:5000; Thermo Fisher Scientifc, Inc., Waltham, MA, USA) at 37°C for 1 h and were detected by enhanced chemiluminescence. The density of bands was quantified using Labworks image acquisition and analysis software (UVP, Fremont, CA, USA).

### 2.8. Cell Apoptosis Assay

Cell apoptosis was evaluated in kidney sections with a terminal deoxynucleotidyl transferase-mediated uridine triphosphate nick end labeling (TUNEL) assay using the Roche Diagnostics kit (Indianapolis, IN, USA). Apoptotic cells were counted in 10 high-power (400×) fields for quantification.

### 2.9. Real-Time PCR Analysis

Total RNA was isolated from renal tissues by Trizol reagent and 2 *μ*g of total RNA was used for synthesis of cDNA by the Reverted First Stand cDNA Synthesis Kit (Thermo Scientific, Waltham, MA, USA) as previously described[22]. Quantitative real-time PCR was performed with LightCycler 480 system (Roche, Pleasanton, CA, USA). The relative mRNA expression levels were normalized to GAPDH and were calculated using the 2^−∆∆Ct^ approach as previously reported [23]. PCR primers are shown in Table 1.

### 2.10. Statistical Analysis

The Graphpad Prism 5 software (La Jolla, CA, USA) was used for statistical analysis and data were presented as mean ± SD. Data are analyzed by one-way ANOVA or Student's t-test. P<0.05 was considered statistically significant.

## 3. Results

### 3.1. Establishment of Cisplatin-Induced Acute Kidney Injury Animal Model

**Figure 1:**
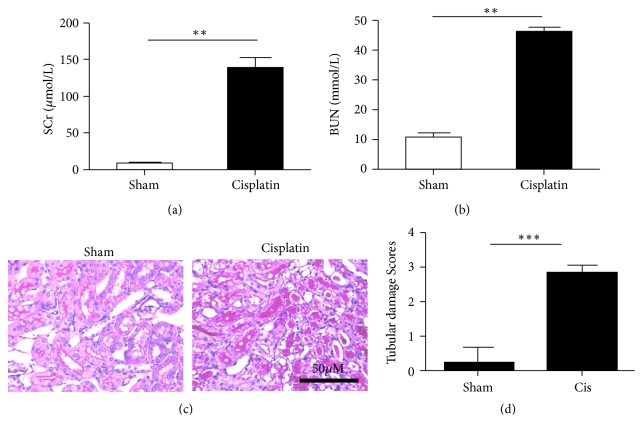
Establishment of cisplatin-induced acute kidney injury (AKI) model in mice. (a) Serum creatinine (Cr) and (b) urea nitrogen (BUN) levels were measured after 22 mg/kg cisplatin injection. (c) PAS staining was conducted on kidney sections. The cisplatin-treated kidneys marked injury after 72 h. (d) Semiquantitative analysis of PAS staining for the severity of renal injury. The values are presented as the means±SEM, n = 8. *∗∗*P < 0.01; *∗∗∗*P < 0.001.

The serum levels of creatinine and blood urea nitrogen (BUN) in mice were measured 72 h after cisplatin treatment, and the renal tissue sections were stained with PAS. Compared with the control group, the blood creatinine and urea nitrogen levels were significantly increased in cisplatin-treated mice (*P*<0.05) (Figures 1(a) and 1(b)). PAS staining revealed that the renal tubules were dilated and loosely arranged and renal tubular epithelial cells exhibited swelling and vacuolar degeneration. Brush border necrosis and exfoliation as well as protein cast formation have been observed, but the glomerulus showed no noticeable variation (Figures 1(c) and 1(d)). All these characteristics indicated that the model was successfully established.

### 3.2. NAC Protects Mice against Cisplatin-Induced AKI

**Figure 2:**
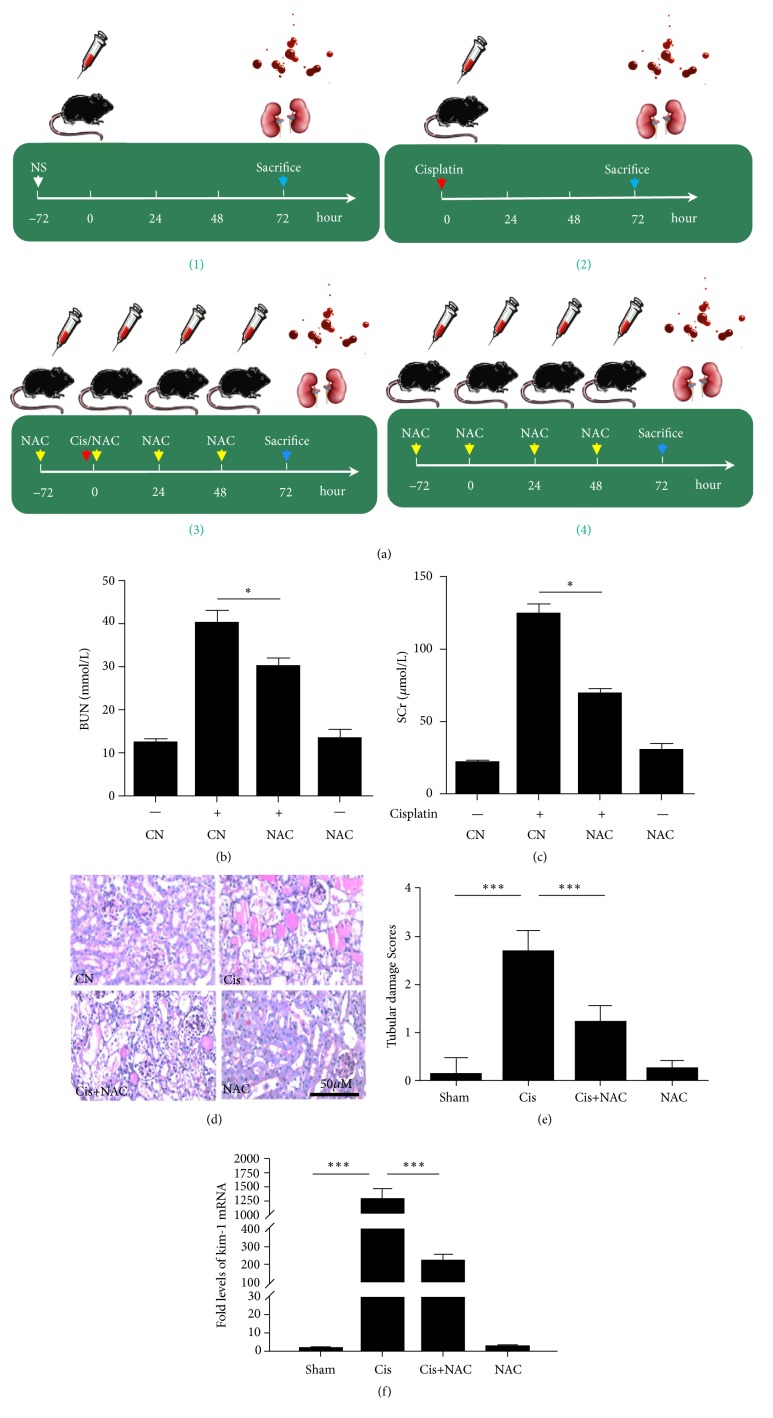
Effects of N-acetylcysteine (NAC) protect mice against Cisplatin-induced AKI. (a) Experimental protocol: C57BL/6 mice received Cisplatin (22mg/kg) by intraperitoneal injection. Animals were allocated to four groups: Control; Cisplatin; Cisplatin+NAC; NAC; (b) Serum creatinine (Cr) and (c) urea nitrogen (BUN) levels of four groups. (d) PAS staining was conducted on kidney sections in each group. (e) Semiquantitative analysis of PAS staining for the severity of renal injury. (f) Real-time PCR was used to measure renal expression of KIM-1. The values are presented as the means±SEM, n = 8. *∗*P < 0.05, *∗∗∗*P < 0.001.

Mice were randomly divided into four groups as follows: (1) saline-treated control group, (2) cisplatin group, (3) cisplatin+N-acetylcysteine group, and (4) N-acetylcysteine group (Figure 2(a)). The results showed that mice treated with cisplatin have higher blood creatinine and urea nitrogen levels compared with control mice. However, the cisplatin treatment performed three days after the NAC pretreatment revealed that the blood creatinine and urea nitrogen levels were significantly decreased (*P*<0.05) (Figures 2(b) and 2(c)). PAS staining of the renal paraffin sections indicated a severe pathological damage of the kidney in the cisplatin-treated group, while the tissue in the control group was normal. Mice pretreated with NAC before the cisplatin treatment showed a significantly milder renal damage compared with the cisplatin-treated group that had not been pretreated. There was no improvement in the only NAC-treated group compared with the control group (Figures 2(d) and 2(e)). The renal KIM-1 level correlated with the renal injury described above (Figure 2(f)).

**Figure 3:**
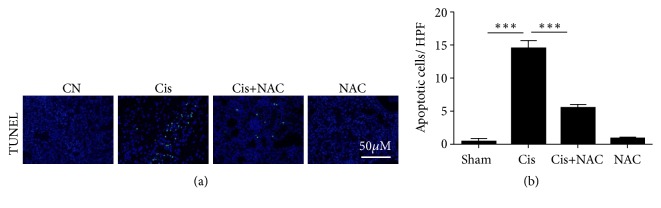
The effect of NAC on apoptotic cells in kidney with TUNEL staining. (a) Apoptosis in the kidney was assessed by the TUNEL assay and typical experiments are presented here. (b) Semiquantitative analysis of TUNEL-positive cells. The values are presented as the means±SEM, n = 8. *∗∗∗*P < 0.001.

To determine the mechanisms underlying the protective effect of NAC against cisplatin-induced AKI, we examined cell apoptosis in the kidney after cisplatin treatment, using a TUNEL assay. After cisplatin treatment, the number of TUNEL-positive cells was clearly increased in the kidneys, whereas mice from Cis+NAC group manifested a marked reduction in TUNEL-positive cells (Figures 3(a) and 3(b)).

### 3.3. NAC Attenuates Oxidative Stress of Kidney in Cisplatin Treated Mice

**Figure 4:**
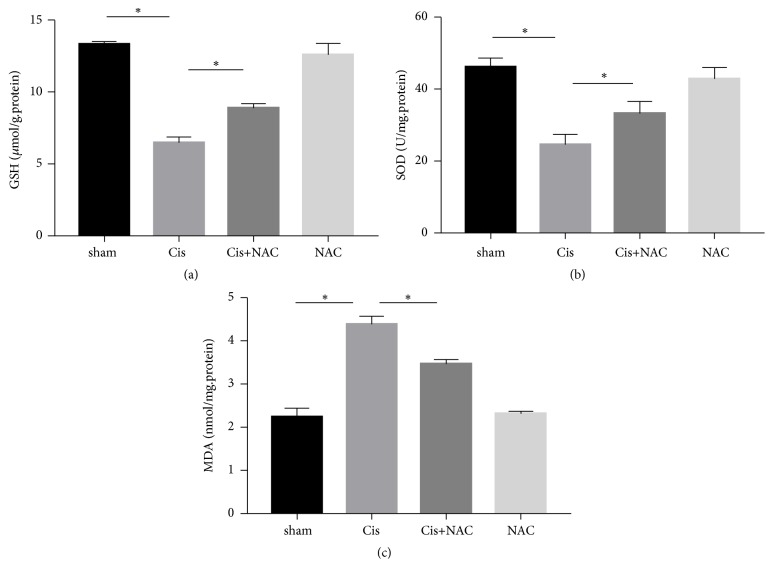
Effects of NAC on the levels of glutathione (GSH) (a); superoxide dismutase (SOD) (b); and malondialdehyde (MDA) (c) in cisplatin-induced AKI. The values are presented as the means±SEM, n = 4. *∗* p < 0.05.

To assess whether NAC has antioxidant effects on cisplatin-induced AKI, we examined some oxidative biochemical parameters. Compared with the control group, the levels of GSH (p < 0.05) and SOD (p < 0.05) were significantly decreased along with an increase in MDA (p < 0.05) in the cisplatin-treated group. In contrast, administration with NAC (50 mg/kg) for three continuous days was markedly reversed these changes (Figure 4).

### 3.4. NAC Decreases Kidney Expression of C5a/C5aR in Cisplatin Treated Mice

**Figure 5:**
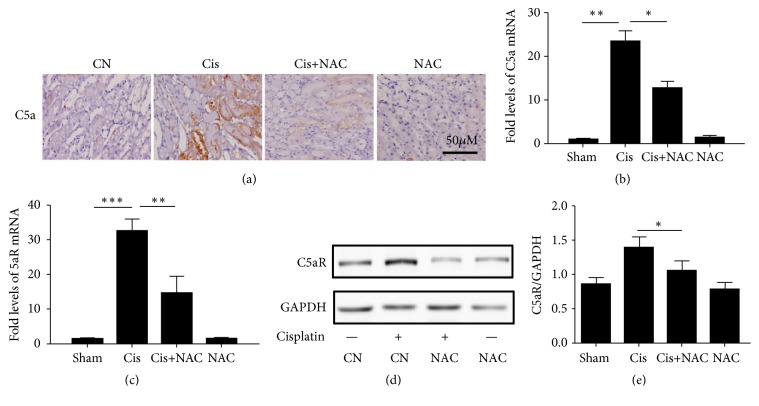
NAC attenuates C5a/C5aR deposition in renal parenchyma. (a) Immunohistochemistry (IHC) showed that the C5a expression was in the CN, Cis, Cis+NAC, and NAC group. The C5a expression was increased in the Cis group and decreased after NAC treatment in the Cis+NAC group; (b and c) Real-time PCR of C5a and C5aR was performed to evaluate the expression level in each group in kidney. (d) the protein level of C5aR in kidney was detected by Western blot. (e) the histogram shows the relative intensity for each marker normalized to GAPDH. The values are presented as the means±SEM, n = 8. *∗*P < 0.05, *∗∗*P < 0.01, and *∗∗∗*P < 0.001.

Histochemical staining revealed that the C5a expression in the cisplatin-treated group was notably increased compared with the control group and this increase could be significantly inhibited by NAC (Figures 5(a) and 5(b)). The NAC group showed no obvious difference in the C5a expression. These results were confirmed by real-time PCR. We further investigated C5aR expression by real-time PCR and western blot. It is noteworthy that C5aR expression was synchronous with C5a (Figures 5(c), 5(d), and 5(e)).

### 3.5. NAC Inhibits the Influx of Neutrophils Expressing C5aR.

**Figure 6:**
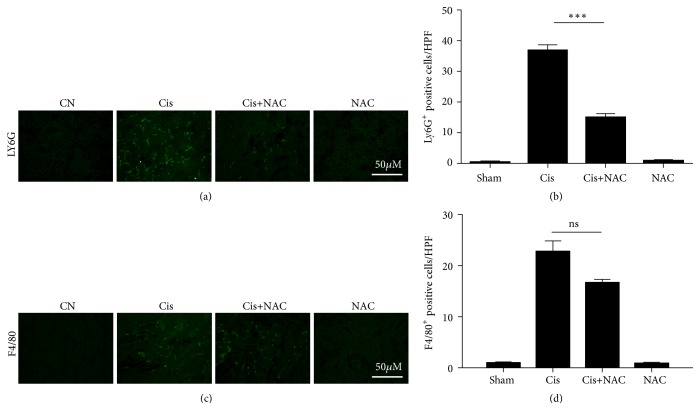
NAC attenuates the cisplatin-induced tubulointerstitial inflammation. (a and b) Immunofluorescence staining of Ly6G was performed to analyze the infiltration of neutrophils; (b) is the quantification of the results shown in (a). (c and d) F4/80 staining was performed to assess the number of macrophages; (c) is the quantification of the results shown in (d). The values are presented as the means±SEM, n =8. *∗∗∗*P < 0.001.

To investigate whether the cisplatin-induced AKI from Cis+NAC group was due to an attenuated inflammatory response, we measured the infiltration of neutrophils and macrophages in the tubulointerstitial region. According to the immunofluorescence results, compared with the negative staining in the sham and NAC groups, mice treated with cisplatin had significant inflammatory cell infiltration, especially neutrophils, while this reaction was markedly attenuated in the Cis+NAC group (Figure 6).

**Figure 7:**
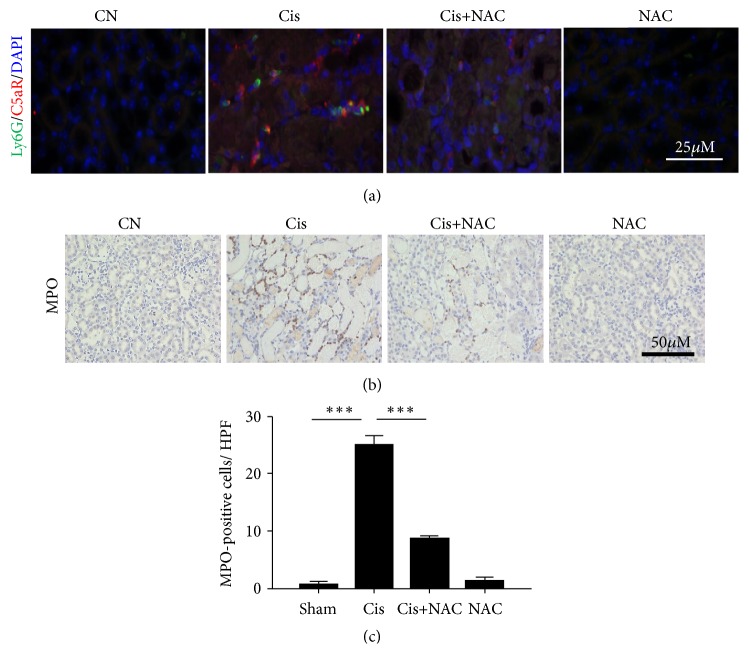
NAC attenuates the cisplatin-induced renal neutrophil infiltration. (a) Immunofluorescence staining showing increased C5aR (red) expression in the interstitium and colocalization with Ly6G (Green). (b) Immunohistochemistry staining of MPO was performed to assess the neutrophil infiltration. (c) Semiquantitative assessment of MPO staining. The values are presented as the means±SEM, n =8. *∗∗*P < 0.01.

Further research found the ly6G^+^ neutrophils coexpressed distinctly with C5aR in the cisplatin-treated group (Figure 7(a)), whereas after NAC treatment, the number of infiltrating neutrophils (MPO^+^ly6G^+^) decreased, suggesting that the production of large amounts of C5a may be the reason for neutrophil infiltration (Figures 7(b) and 7(c)). No significant difference was observed in the F4/80^+^ macrophages between four groups.

### 3.6. NAC Down-Regulates the Expression of Cisplatin-Induced Inflammatory Factors

**Figure 8:**
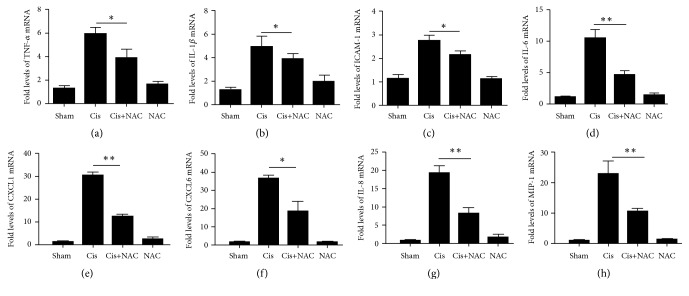
NAC can inhibit the release of chemokines in cisplatin injured mice. Real-time PCR showing relative renal mRNA levels of TNF-*α*(a), IL-1*β*(b), ICAM-1(c), IL-6(d), CXCL1(e), CXCL6 (f), IL-8 (g), and MIP-1(h) in each group in kidney. The values are presented as the means±SEM, n =8. *∗*P < 0.05; *∗∗*P < 0.01.

As shown in Figure 8, the expressions of the inflammatory factor TNF-*α*, IL-1*β*, ICAM-1, IL-6, CXCL1, IL-8, CXCL6, and MIP-1 increased in the cisplatin-induced kidney model, while these expressions decreased in the cisplatin/NAC-treated group, suggesting that NAC has a protective effect on the kidney. Groups treated with NAC showed no significant difference compared with the control.

## 4. Discussion

Cisplatin is one of the first-line drug against many cancers and has been widely used in chemoradiation therapy. But its application is limited by the severe nephrotoxicity, which particularly affects the proximal tubule epithelial cells due to accumulation and selective endocytosis of aminoglycosides [24]. Multiple mechanisms, for example, oxidative stress, vascular injury, inflammation, and proximal tubular necrosis, have been associated with the progression of cisplatin-induced AKI [25]. In our study, we report, for the first time, the NAC was ameliorated cisplatin-induced renal toxicity by inhibiting the C5a expression without compromising its antioxidant activity.

NAC is a thiol-containing antioxidant, which hinders cisplatin from removing glutathione or accumulating the peroxide. It directly reduces the amount of reactive oxygen species (ROS) and protects against kidney damage [26, 27]. The cell apoptosis pathway mediated by oxidative stress and P53 may participate in cisplatin-induced acute renal failure. Inhibiting oxidative stress and deactivating the P53 protein by NAC can alleviate the cisplatin-induced renal toxicity [28–30]. Previous studies pay more attention on the oxidative stress in the process of cisplatin induced kidney damage. Our work showed similar results that cisplatin can cause a significant decrease in the contents of SOD and GSH and an increase in the content of MDA in the kidneys. NAC, as the ROS specific inhibitor scavenger, can enhance the activities of antioxidant enzymes and GSH levels as well as the inhibited MDA formation [31, 32]. These data suggested that NAC ameliorated oxidative damage in kidney tissues by increasing antioxidant enzyme activity. The anti-inflammatory property of NAC is rarely studied.

Although cisplatin directly causes drug accumulation and subsequent cytotoxicity in the proximal tubules, cytokines and chemokines also contribute in exacerbating kidney injury [33]. In particular, TNF-*α* and IL-1*β* have a pivotal role on cisplatin renal injury, and little attention has been paid to the chemokines [34–36]. Hence, we investigate changes in the chemokines in the kidney after cisplatin injury. We found that CXCL1, CXCL6, and MCP-1 as chemoattractant for inflammatory cells markedly increased in the whole kidney after cisplatin administration. Interestingly, neutrophil number as evaluated with macrophages increased with cisplatin administration in kidney, especially neutrophils, while this reaction was markedly attenuated after NAC treatment. Neutrophils have been reported to promote kidney injury and inflammation after cisplatin-induced AKI. Downregulation of CXCL-1 and CXCL-2 may be secondary to mitigation in IR injury and neutrophil infiltration, as infiltrated neutrophils express significant levels of interleukin-17A, CXCL-1, and CXCL-2 expression [37, 38].

The complement system plays a very important role in the innate immunity and can be activated by three pathways: the classical pathway, the alternative pathway, and the lectin pathway. Each of these ways can cause the cleavage of C5 into C5a and C5b, two anaphylatoxins that later form the first part of the complement membrane attack complex [39, 40]. According to our previously published paper, we had demonstrated that complement C3 facilitates IL-17A production in T cells, contributing to the development of renal inflammation and fibrosis [41]. In renal IR injury, C5a, binding with C5aR, played an important role in the initiation and regulation of inflammatory responses [42]. In glomerulonephritis model, C5a acting through its cognate receptor C5aR participated in immune complex deposition and glomerular inflammation. Simultaneously, accumulating evidence showed that C3aR/C5aR deficiency resulted in reduced proteinuria and attenuated histological injury by inhibiting expression of cytokines and chemokines in an IgAN mouse model. In present study, we found that the C5a was constitutively expressed throughout the kidney in the cisplatin-treated group, from which it was released by ly6G^+^neutrophils. The expression of C5a and C5aR and infiltrating ly6G+ neutrophils were reduced by NAC treatment.

The limitation of this current study is that we have no firm evidence to support NAC itself being present in the target tissue. Different plasma concentration might elicit a variety of biological functions. NAC is a direct antioxidant that interacts with the hydroxyl radical (·OH), nitrogen dioxide (·NO2), carbon trioxide ion (CO3^·−^), and other compounds [43]. And NAC might even just act to chemically modify cisplatin and detoxify it directly in cisplatin-caused renal toxicity. Apart from its effect as a direct antioxidant, NAC also functions as an indirect antioxidant. If the plasma concentration is quite low, NAC maybe exert indirect antioxidative properties by maintaining GSH levels in the intracellular environment[10]. It is noteworthy that high doses of NAC (400mg/kg) are used to prevent toxicity to acetaminophen poisoning. Another literature also reported that high-dose intravenous rapid injection of NAC can exert thrombolytic effect by reducing the size of von Willebrand Factor (VWF) multimers [44]. A Double-Blind, Randomized, Placebo-Controlled Trial (HIACE) study demonstrated that high-dose NAC (600 mg bid) could reduce COPD exacerbations and improve small airway function [45]. It is a real shame that NAC was not measured in plasma in this study. That would give insights into the relevance to oral/i.v. opportunities in patients and would also provide some information as to whether the effects are direct and/or off-target or wholly mediated by incorporation into glutathione. More specific regulatory mechanisms of the inflammatory response by NAC through the complement requires further investigation.

In conclusion, we have provided evidence demonstrating that pretreatment with NAC can protect the kidney from cisplatin-induced AKI by inhibiting the C5a-C5aR pathway and the downstream inflammatory reaction. This research helps us to determine the role of the complement in AKI and to obtain a better understanding of the anti-inflammatory effect of NAC, which may provide a new clinical treatment for AKI.

## Figures and Tables

**Table 1:** Primers used to amplify cDNAs for mice.

*Primers*	Sequence (Sense/Antisense)
TNF-*α*	CTGAACTTCGGGGTGATCGG
	GGCTTGTCACTCGAATTTTGAGA

ICAM-1	GAGGAGCAGTTGCGGTCTG
	TCCTGGTATTGAGGGTGGG

IL-1*β*	AGCTTCCTTGTGCAAGTGTCT
	GACAGCCCAGGTCAAAGGTT

C5aR	GGGTCCGTTCCAGAAAATGT
	GACAAGGGACCGGGGTCCAA

GAPDH	AGGTCGGTGTGAACGGATTTG
	GGGGTCGTTGATGGCAACA

## Data Availability

The data used to support the findings of this study are included within the article.
